# Early non-invasive epigenetic approach for assessing trisomy risk in maternal plasma

**DOI:** 10.3389/fmed.2026.1704017

**Published:** 2026-03-04

**Authors:** Tridiv Katiyar, Medha Srivastava, Suman Mishra, Swasti Tiwari, Shubha Phadke

**Affiliations:** 1 Department of Molecular Medicine & Biotechnology, Sanjay Gandhi Post Graduate Institute of Medical Sciences (SGPGIMS), Lucknow, Uttar Pradesh, India; 2 Department of Medical Genetics, Sanjay Gandhi Post Graduate Institute of Medical Sciences (SGPGIMS), Lucknow, Uttar Pradesh, India

**Keywords:** trisomy, epigenetic markers, extracellular vesicles derived DNA, free fetal DNA, methylated cell-free DNA

## Abstract

**Background:**

Karyotyping is the standard confirmatory test for identifying chromosomal abnormalities, such as Trisomy 21, which requires amniotic fluid from pregnant women. The present study investigated the potential of methylated cell-free DNA (mcf-DNA) and methylated extracellular vesicle-derived DNA (mev-DNA) as an early, non-invasive epigenetic approach, specifically focusing on fetal-specific methylated regions (FSMRs) of *RASSF1A, ERG,* and *UMODL1 (U1 and U2)* to assess Trisomy 21 risk in maternal plasma.

**Methods:**

Blood samples were collected from pregnant women (*n* = 120) between 10th and 24th weeks of gestation who were at higher risk for Trisomy 21. Of the 120 women, 3 were found to be positive for Trisomy 21 through karyotyping method. Moreover, mcf-DNA and mev-DNA were isolated from Trisomy-positive and age-matched healthy pregnant women (*n* = 8) and non-pregnant women (*n* = 8). FSMRs were analyzed using qPCR to compare the cycle threshold (Ct) values. Gene copy number analysis was performed using the plasmid standards method to assess Trisomy 21 detection sensitivity.

**Results:**

Trisomy pregnancies had significantly lower mean Ct values for *RASSF1A* and *UMODL1 (U1* and *U2)* in both mcf-DNA and mev-DNA than healthy pregnancies, which was further confirmed by higher copy numbers in trisomy pregnancies than in healthy pregnancies. Moreover, the gene copy number in mcf-DNA was significantly higher than that in mev-DNA for the *RASSF1A, ERG,* and *UMODL1 (U1* and *U2)* genes in trisomy pregnancies.

**Conclusion:**

This pilot study demonstrates the feasibility of using mcf-DNA and mev-DNA for detecting Trisomy 21-associated fetal methylation signatures in maternal plasma. While consistent with earlier findings, these results validate the applicability of methylated DNA immunoprecipitation (MeDIP)-based qPCR methylation assays in a North Indian cohort and support their potential integration into population-specific non-invasive prenatal screening strategies.

## 1 Introduction

During pregnancy, the placenta is a significant source of free fetal DNA (ff-DNA) in maternal blood, whereas maternal blood cells are the primary contributors to maternal plasma DNA. Due to the limited availability of fetal-fraction, a cost-effective, non-invasive method is required for prenatal diagnosis. In this respect, the discovery of cell-free fetal DNA (cff-DNA) in maternal plasma opens new possibilities for non-invasive prenatal diagnosis (1–3). Some studies have shown that the concentration of cff-DNA in maternal plasma ranges between 10 and 13% of the total cell-free DNA (cf-DNA). During pregnancy, cf-DNA is released into maternal plasma from placental trophoblast cells, which contain a portion of cff-DNA. Moreover, cff-DNA can be detected from 4 weeks of gestation and is cleared from the maternal circulation within 2 h after delivery (2, 4). Therefore, cff-DNA in maternal plasma has been used for non-invasive prenatal testing of fetal chromosomal anomalies, fetal rhesus D (RhD) status, paternally inherited genetic diseases, and sex-linked disorders (5, 6).

In addition, the rapidly growing field of extracellular vesicles (EVs) research offers valuable biological signatures, as biomolecules remain stable within lipid-encapsulated structures. Consequently, there is strong evidence supporting the use of EV components as a non-invasive diagnostic tool (7). EVs function as intercellular communicators in various body fluids and contain nucleic acids, proteins, and lipids. During pregnancy, placental trophoblasts release EVs into the maternal circulation, with their concentrations increasing as gestation progresses. Some studies have reported that DNA present in EVs serves as a useful marker for disease monitoring (8, 9). In this study, we aimed to evaluate a fetal-specific, non-invasive epigenetic approach using EVs to assess the risk of Trisomy 21.

Previous approaches using ff-DNA for non-invasive prenatal diagnosis have encountered several challenges affecting their accuracy and effectiveness. While next-generation sequencing technologies have been explored, their high costs and limited accessibility in basic diagnostic laboratories restrict their widespread use (10, 11). One method utilizes methylation-sensitive restriction enzymes to eliminate hypomethylated maternal DNA, allowing direct polymerase chain reaction analysis of fetal DNA. However, this technique is constrained by the requirement for differentially methylated regions with specific restriction sites, thereby reducing the number of suitable regions for analysis (12). Another commonly used method involves treating DNA with sodium bisulfite, which converts unmethylated cytosines to uracil, while leaving methylated cytosines unchanged. Although effective, the harsh conditions of bisulfite treatment can cause significant DNA degradation, complicating downstream analyses (13, 14). To address these limitations, our study employed the MeDIP technique, which enriches and isolates methylated DNA by targeting the methyl-CpG-binding domain (MBD) protein, enabling the investigation of DNA methylation patterns on chromosome 21. This approach offers a more targeted and efficient alternative for detecting fetal DNA methylation in prenatal screening. Certain genes are methylated in the fetal portion, whereas their maternal counterparts are typically unmethylated, and these regions have been reported as FSMRs (15). We hypothesize that examining these FSMRs on chromosome 21 will enable effective differentiation between normal and Trisomy 21 cases based on their methylation status.

The placenta releases cff-DNA into the maternal circulation, and the methylation status of the *RASSF1A* gene makes it a valuable marker for detecting and quantifying cff-DNA in the maternal blood, which is especially useful for non-invasive prenatal testing of genetic abnormalities or chromosomal anomalies (1, 4, 16). In this study, we conducted a pilot investigation to reproduce and refine established MeDIP-qPCR-based approaches for detecting FSMRs within the *ERG* and *UMODL1 (U1* and *U2)* loci on chromosome 21, alongside the established fetal fraction marker *RASSF1A* on chromosome 3. The study aimed to validate the technical feasibility and analytical performance of mcf-DNA and mev-DNA analyses for early, non-invasive assessment of Trisomy 21 risk in a North Indian cohort.

## 2 Materials and methods

### 2.1 Study subjects

A sample of 5 mL of peripheral venous blood was collected in ethylene diamine tetra-acetic acid (EDTA) tubes from a prospective cohort of non-pregnant women (*n* = 25) and pregnant women (*n* = 120) between the 10th and 24th weeks of pregnancy, who attended routine antenatal visits at the OPD of the Medical Genetics Department, SGPGIMS Hospital, after providing written informed consent. The present study was reviewed and approved by the Human Ethics Committee of SGPGIMS, Lucknow (IEC: 2022-44-SRF-EXP-46). Gestational age was estimated based on the first day of the last menstrual period and confirmed by transvaginal ultrasound at recruitment (i.e., ∼6–8 weeks). Plasma was isolated within 2 h of collection by centrifugation at 3,000 rpm for 10 min at 4 °C and then stored into aliquots at −80 °C until further processing. Aliquots were used to isolate cf-DNA and extracellular vesicle-derived DNA (ev-DNA), which were further processed for expression analysis.

### 2.2 Inclusion criteria

The obstetrical history and physical findings of all pregnant women enrolled in the study indicated the absence of any previous infections of the uterus or amniotic cavity, hypertension, gestational diabetes, or preeclampsia, confirming a healthy pregnancy. Controls (non-pregnant and healthy pregnant women) who lived in the same geographical area, who had a similar socioeconomic status, who belonged to the same ethnicity (Indo-European community), and who were of comparable age were included in the study.

### 2.3 Exclusion criteria

Pregnant women with multiple pregnancies, chronic illnesses, or a history of smoking were excluded from this study.

### 2.4 Biochemical screening for aneuploidies

Biochemical screening for aneuploidies was conducted using dual-, triple-, and quadruple-marker tests, and Prisca software was employed to calculate the risk of aneuploidies (Trisomy 13, 18, and 21). In cases where the risk of aneuploidies was deemed high based on these biochemical markers, karyotyping via amniocentesis was performed for chromosomal abnormalities between the 15th and 25th weeks of pregnancy. Women were categorized as high or low risk based on the results of biochemical screening and karyotyping and subsequently included in the study (if they had confirmed chromosomal abnormalities or normal chromosomal findings). Based on biochemical screening and karyotyping, of the 120 participants, 3 were Trisomy 21-positive, 89 had normal karyotyping results, 15 samples required repeat sample collection for karyotyping, and 13 samples were lost to follow-up.

### 2.5 EVs isolation and characterization

EVs were isolated from 1 mL of blood plasma from pregnant and non-pregnant women using differential ultracentrifugation. In the first step, plasma from each participant was diluted fivefold in PBS, followed by ultracentrifugation at 126,000 *g* for 2 h at 4 °C. After centrifugation, the supernatant was discarded, and the resulting EV pellets were suspended in 50 μL of PBS for downstream analysis (17). EVs were characterized for the presence of exosomal markers CD63 using magnetic-coated CD63-Dynabeads (Thermo Fisher Scientific, Waltham, Massachusetts, United States) according to the manufacturer’s instructions and analyzed on a Beckman Coulter, Inc. in the United States, DxFLEX Flow Cytometer. The data were analyzed using CytExpert v2.0 software (Beckman Coulter). Particle size and concentration of EVs were determined by Nanotracking Analysis (NTA) using the NanoSight NS300 (Malvern Instruments Ltd., United Kingdom) at the Central Research facility of the Indian Institute of Toxicological Research, Lucknow, following the protocol standardized by our laboratory (17).

### 2.6 Isolation of cf-DNA and ev-DNA from maternal plasma

One milliliter of plasma from each participant was thawed prior to extraction of cf-DNA and ev-DNA. cf-DNA was isolated from plasma, while ev-DNA was isolated from total EVs obtained from blood plasma, which was first treated with 1 unit of DNase I (NEB, United States) for 1 h at 37 °C to remove any cf-DNA bound to the surface of the vesicles. DNase I was then deactivated using 10 mM EDTA. Both cf-DNA and ev-DNA were then isolated from all studied subjects using the QIAamp DSP Virus Kit (Qiagen, CA, United States) according to the manufacturer’s protocol, with minor modifications.

### 2.7 Enrichment of methylated DNA

Methylated DNAs (mcf-DNA and mev-DNA) were isolated and enriched from cf-DNA and ev-DNA in maternal plasma using the MethylMiner™ Methylated DNA Enrichment Kit (Invitrogen, Carlsbad, United States; Catalog no. ME10025). Methylated and non-methylated DNA control duplexes, provided in the MethylMiner™ Methylated DNA Enrichment Kit, were included to validate the enrichment efficiency. The methyl-CpG-binding domain of the human MBD protein was bound to 10 μL of Dynabeads M-280 Streptavidin. This mixture was incubated at room temperature for 30 min with gentle agitation to ensure efficient binding. One microgram of cf-DNA and ev-DNA was added to separate tubes containing the MBD magnetic bead conjugates and incubated at room temperature for 1 h with gentle rotation to allow the methylated DNA to bind to the MBD-bead conjugates. The bound mcf-DNA and mev-DNA were then eluted from the MBD-bead conjugates using a multi-fraction elution buffer containing 200–2000 mM NaCl. Finally, the eluted mcf-DNA and mev-DNA were concentrated using ethanol precipitation.

### 2.8 Quantification of mcf-DNA and mev-DNA through real-time quantitative PCR

For the absolute quantification of FSMRs, DNA copy numbers were calculated using the method described by Lee et al. (18). Copy number quantification was achieved by interpolating Ct values against a standard curve (Ct values vs. log₁₀ copy number) generated from serial dilutions of gene-specific plasmid standards (GenScript). The resulting copy numbers per reaction were normalized to copies per nanogram (ng) of input DNA (Supplementary Table S1). The performance of the qPCR assay was confirmed through standard curve analysis, which demonstrated amplification efficiencies ranging from 93 to 110%, with *R*^2^ values greater than 0.98. In brief, all mcf-DNA and mev-DNA from the study groups were quantified using real-time qPCR with single-plex SYBR Green PCR assays in 96-well optical plates on a BIO-RAD CFX96 Real-Time PCR System. Primers were used to amplify the FSMRs listed in Table 1. All qPCR amplification reactions were performed in duplicate in a total volume of 20 μL. ΔCt normalization was applied using *RASSF1A* as the internal reference gene to correct for inter-sample variation. The reaction mixture contained 10 μL of 1 × SYBR Green PCR master mix (Takara Bio, Japan), 10 pmole of each gene-specific primer, methylated DNA samples, and nuclease-free water to make up the remaining volume. PCR conditions were as follows: initial denaturation at 95 °C for 5 min, followed by 40 cycles consisting of denaturation at 95 °C for 5 s, combined annealing/extension at 58–60 °C for 30 s, and 72 °C for 1 min.

**Table 1:** Primers sequence used for amplification sites of fetal specific epigenetic regions.

Gene locus	Primer sequences	Location of primers
RASSF1A	FP: 5′- -GAG CCT GAG CTC ATT GAG CTG − 3’	Chromosome 3
	RP: 5′- ACC AGC TGC CGT GTG G − 3’	
ERG	FP: 5′- ACA GCT GAA GCT GGG CCG − 3’	Chromosome 21
	RP: 5′- ATG GCA GAT GCC ATC AGA CG - 3′	
UMODL1 (U1)	FP: 5′- ACC CCA CGT GCA CTG AGC G − 3’	Chromosome 21
	RP: 5′- CAC TTC TGC CCT CTG CCC G − 3’	
UMODL1 (U2)	FP: 5′- GGT GCA CGC AAG GAG CTA TCG-3’	Chromosome 21
	RP: 5′- TGG TGC ACA CGG CTG CTT CCG − 3’	

### 2.9 Statistical analysis

Data were analyzed using GraphPad Prism software (version 9.0). Mean Ct values and corresponding standard deviations were calculated for each group to assess data variability. To determine statistical significance, unpaired *t*-tests were performed to compare independent groups: non-pregnant women, healthy pregnant women, and Trisomy pregnancies. All tests were two-tailed, and a *p*-value < 0.05 was considered statistically significant.

## 3 Results

### 3.1 Maternal serum biochemistry and demographics

The study population included women aged 25–40 years and was divided into three groups: non-pregnant women (*n* = 8), healthy pregnant women (*n* = 8) with normal biochemical screening and karyotyping results, and women with Trisomy 21 pregnancies (*n* = 3). The biochemical and demographic characteristics of the study participants are summarized in Table 2.

**Table 2:** Biochemical and demographic variables of the study participants.

Demographics	Healthy reference range	Healthy pregant women	Pregnant women with trisomy (+) cases
		Mean (SD)	Mean (SD)
Age at time of enrolments (years)	--	29.87 (3.80)	33 (3.79)
Gestational age at the time of sample collection (weeks)	--	20.57 (2.49)	21.46 (7.36)
Biochemicals screening for aneuplodies			
Gestational age for 1–2 parameters (weeks)	10–13	12.7 (0.7)	--
(1) Pregnancy-associated plasma protein A (PAPP-A) (mlU/mL)	0.1–32.3	4.08 (1.87)	--
(2) Free beta-human chorionic gonadotrophin fb-hCG (ng/mL)	5.6–388.7	37.37 (13.06)	--
Gestational age for 3–6 parameters (weeks)	14–22	15.07 (3.71)	20.25 (1.20)
(3) Alpha-fetoprotein (AFP) (ng/mL)	5.4–501	63.33 (8.88)	36.75 (14.21)
(4) Unconjugated estriol (UE3) (ng/mL)	0–11	1.04 (0.81)	1.53 (1.05)
(5) Human chorionic gonadotropin (hCG) (mlU/mL)	2,223–200,230	33,301 (17,364)	26,363 (4199)
(6) Inhibin-A (Inh-A) (pg/mlL)	236.78–373.33	280.9 (111.34)	554 (120)
Risk for trisomy 21	<1:250	<1:10,000	>1:50
Risk for trisomy 18	<1:100	<1:10,000	<1:10000
Risk for neural tube defects (NTD) (MoM AFP)	AFP MoM <2:5	0.98 (0.46)	0.52 (0.27)

Data are represented as mean (SD).

### 3.2 EVs characterization

#### 3.2.1 NTA analysis

**Figure 1:**
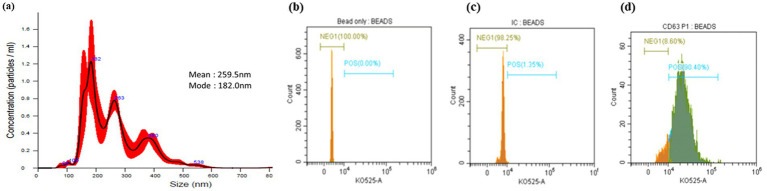
Isolation and characterization of total extracellular vesicles (EVs) from maternal blood plasma. Size distribution and concentration of the EVs analyzed by **(a)** nanoparticle tracking analysis (NTA). Flow cytometry analysis of CD63 expression on the surface of EVs from blood plasma samples, captured on anti-human CD63 magnetic beads. Histogram showing CD63 expression in plasma EVs samples: **(b)** unstained bead only; **(c)** EVs captured on anti-human CD63 bead, incubated with an isotype control and stained with the detection antibody; **(d)** EVs captured on anti-human CD63 beads, incubated with an anti-CD63 primary antibody, and stained with the detection antibody.

NTA showed that the average particle size ranged from 182.0 nm to 259.5 nm. The modal size distribution indicated a predominant population of small EVs in healthy pregnancy samples (Figure 1a).

#### 3.2.2 Flow cytometry analysis

Flow cytometry analysis assessed the expression of the EV-specific marker CD63, confirming the presence of exosomes in the isolated EVs. Histogram data demonstrated CD63 expression in unstained beads, stained EV-bead complexes incubated with an isotype control, and stained EV-bead complexes incubated with an anti-CD63 antibody (Figures 1b–d). Figure 1d shows that approximately 90.4% of EV–bead complexes were positive for CD63, indicating high purity of the isolated vesicles. NTA measured an EV concentration of 5.93 × 10^7^ ± 1.41 × 10^7^ particles/mL. After correction for CD63^+^ purity, this corresponded to an effective concentration of 5.36 × 10^7^ ± 1.28 × 10^7^ CD63^+^ EVs per mL of plasma.

### 3.3 Quantification of mcf-DNA and mev-DNA

**Figure 2:**
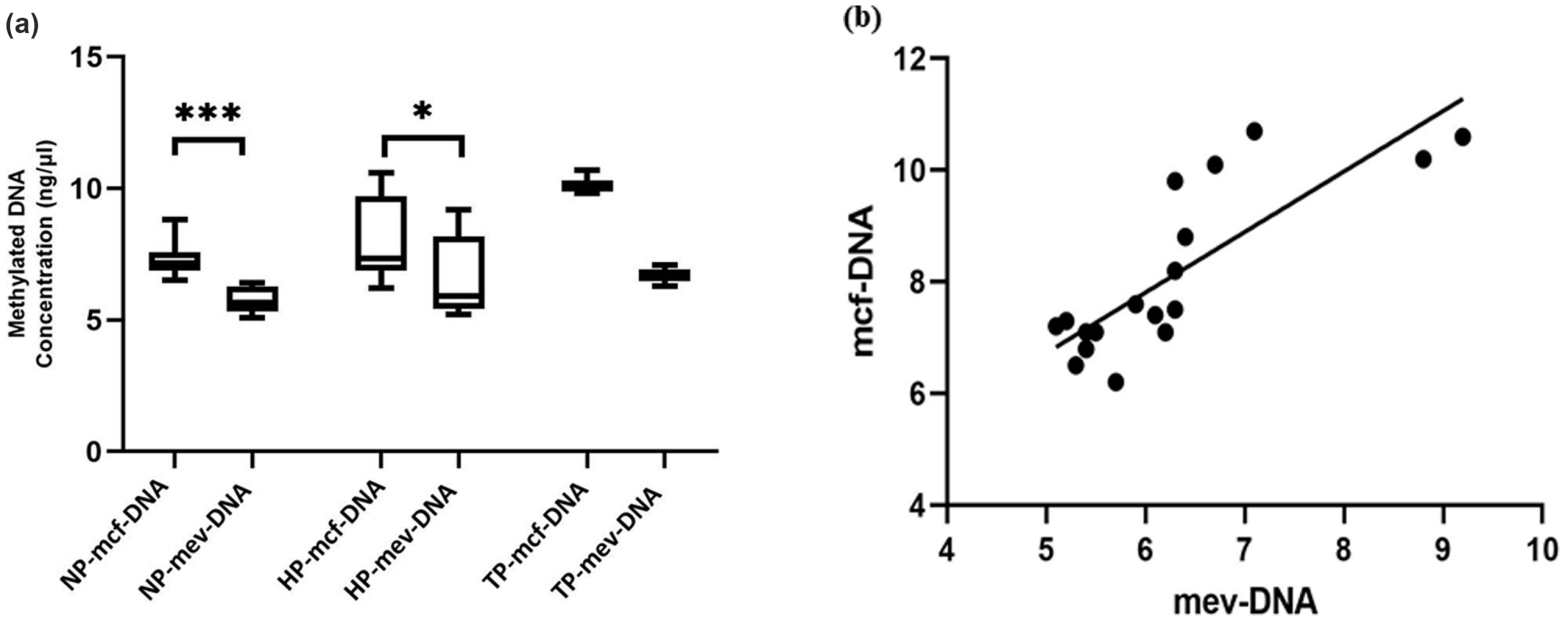
Concentrations of methylated cell-free DNA (mcf-DNA) and methylated extracellular vesicle-derived DNA (mev-DNA) in non-pregnant women (NP), healthy pregnancies (HP), and women with trisomy pregnancies (TP). **(a)** The box plot compares the concentrations of mcf-DNA and mev-DNA (*n* = 8). **(b)** The scatter plot shows the correlation between mcf-DNA and mev-DNA (Pearson correlation coefficient, *r* = 0.81; coefficient of determination, *R*^2^ = 0.668; *p* < 0.0001).

The box plot data compare the mcf-DNA and mev-DNA levels among all study participants. Mcf-DNA concentrations were significantly higher than mev-DNA concentrations in both non-pregnant and healthy pregnant women (*p* < 0.05), but the difference was not significant in women with trisomy (Figure 2a). The scatter plot shows a positive correlation between mcf-DNA and mev-DNA in all studied subjects (coefficient of determination *R*^2^ = 0.668; *p* < 0.0001; Figure 2b). Furthermore, quantitative assessment of enrichment efficiency revealed that approximately 5.63 to 7.88% of total EV-derived DNA was successfully recovered as methylated DNA after enrichment, indicating the effective isolation of the methylated fraction.

### 3.4 qPCR analysis of FSMRs reveals differences in Ct values between mcf-DNA and mev-DNA

**Figure 3:**
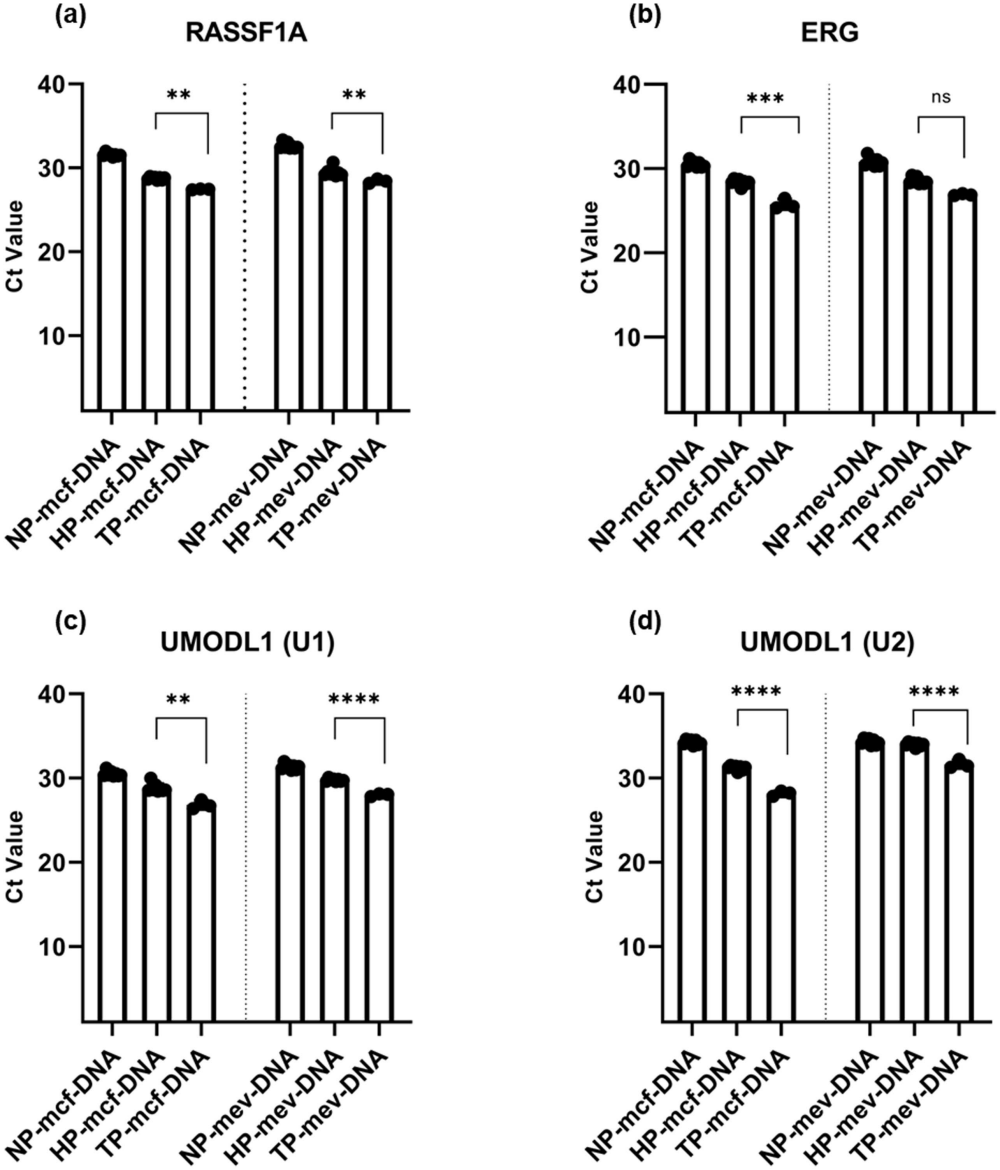
Ct values of fetal-specific methylated regions (FSMRs) in methylated cell-free DNA (mcf-DNA) and methylated extracellular vesicle-derived DNA (mev-DNA) among non-pregnant women (NP), healthy pregnancies (HP), and women with trisomy pregnancies (TP): **(a)** Ct values for the *RASSF1A* gene; **(b)** Ct values for the *ERG* gene; **(c)** Ct values for the *UMODL1 (U1)* gene; **(d)** Ct values for the *UMODL1 (U2)* gene.

The qPCR analysis revealed that both mcf-DNA and mev-DNA showed significantly higher methylation levels in Trisomy pregnancies compared to healthy pregnancies. For the *RASSF1A* methylation gene, the mean Ct value of mcf-DNA was significantly lower in Trisomy pregnancies (27.42 ± 0.50) compared to healthy pregnancies (28.75 ± 0.170), indicating higher levels of *RASSF1A* methylation in Trisomy pregnancies. Similarly, mev-DNA also showed significantly lower mean Ct values in Trisomy pregnancies (28.42 ± 0.255) compared to healthy pregnancies (29.46 ± 0.520; Figure 3a).

For the *ERG* methylation gene, the mean Ct value of mcf-DNA in Trisomy pregnancies (25.72 ± 0.493) was significantly lower compared to healthy pregnancies (28.37 ± 0.35), indicating elevated *ERG* methylation in Trisomy pregnancies. Moreover, the mean Ct value of mev-DNA was also lower in Trisomy pregnancies (26.89 ± 0.13) than healthy pregnancies (28.52 ± 0.38; Figure 3b).

For the *UMODL1 (U1)* gene, the mean Ct value of mcf-DNA was lower in Trisomy pregnancies (26.78 ± 0.429) than healthy pregnancies (28.82 ± 0.51). Moreover, mev-DNA for *UMODL1 (U1)* showed significantly lower mean Ct values in Trisomy pregnancies (27.98 ± 0.178) than healthy pregnancies (29.77 ± 0.163), showing a significant increase in *UMODL1 (U1)* methylation level (Figure 3c).

Further analysis of the *UMODL1 (U2)* gene revealed significantly lower mean Ct values for both mcf-DNA (28.16 ± 0.289) and mev-DNA (31.64 ± 0.465) in Trisomy pregnancies than those in healthy pregnancies (31.20 ± 0.271 for mcf-DNA and 33.98 ± 0.23 for mev-DNA), indicating higher methylation levels. Notably, within Trisomy pregnancies, the mean Ct value for mcf-DNA (28.16 ± 0.289) was much lower than that for mev-DNA (31.64 ± 0.465), suggesting a higher methylation profile in mcf-DNA (Figure 3d).

### 3.5 Gene copy number analysis of FSMRs reveals differences in copy numbers between mcf-DNA and mev-DNA

**Figure 4:**
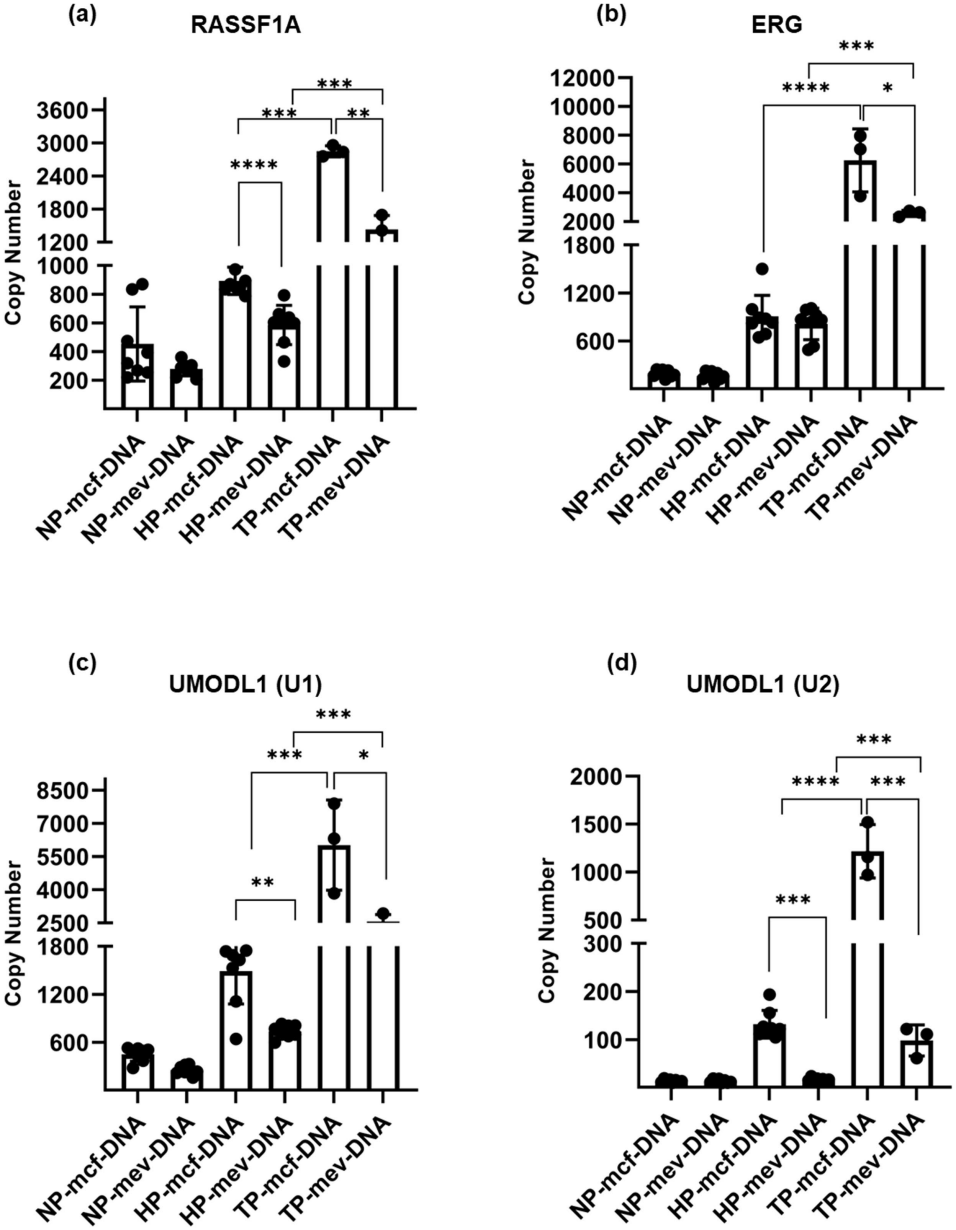
Gene copy number of fetal-specific methylated regions (FSMRs) in methylated cell-free DNA (mcf-DNA) and methylated extracellular vesicle-derived DNA (mev-DNA) among non-pregnant women (NP), healthy pregnancies (HP), and women with trisomy pregnancies (TP): **(a)** copy number of the *RASSF1A* gene; **(b)** copy number of the *ERG* gene; **(c)** copy number of the *UMODL1 (U1)* gene; **(d)** copy number of the *UMODL1 (U2)* gene.

The gene copy number analysis of the *RASSF1A* gene showed that mcf-DNA copy numbers were significantly higher in Trisomy pregnancies (2849.84 ± 101.33) than in healthy pregnancies (892.90 ± 95.12). Similarly, mev-DNA copy numbers were also significantly higher in Trisomy pregnancies (1430.63 ± 256.31) than in healthy pregnancies (585.44 ± 136.91). Notably, within Trisomy pregnancies, mcf-DNA copy numbers were substantially higher than those of mev-DNA (Figure 4a).

Further analysis of the *ERG* gene also revealed significantly higher mcf-DNA copy numbers in Trisomy pregnancies (6253.18 ± 2190.91) than in healthy pregnancies (905.90 ± 264.87). A similar pattern was observed for mev-DNA, with significantly higher copy numbers in Trisomy pregnancies (2571.90 ± 217.27) than in healthy pregnancies (812.29 ± 196.18). Additionally, our results showed that mcf-DNA copy numbers were significantly higher than those of mev-DNA in Trisomy pregnancies (Figure 4b).

For the *UMODL1 (U1)* gene, copy number analysis revealed significantly higher mcf-DNA copy numbers in Trisomy pregnancies (6,106.679 ± 1891.00) than in healthy pregnancies (1,491.10 ± 410.88). Similarly, mev-DNA copy numbers were significantly higher in Trisomy pregnancies (2,916.05 ± 319.49) than in healthy pregnancies (734.58 ± 81.18). Furthermore, mcf-DNA consistently exhibited higher copy numbers than mev-DNA in both Trisomy and healthy pregnancies (Figure 4c).

Finally, the analysis of the *UMODL1 (U2)* gene revealed significantly higher mcf-DNA copy numbers in Trisomy pregnancies (1,216.94 ± 277.80) than in healthy pregnancies (131.98 ± 28.89). Similarly, mev-DNA copy numbers were significantly higher in Trisomy pregnancies (98.10 ± 32.28) than in healthy pregnancies (17.29 ± 3.17) (Figure 4d). Consistently, our data showed that mcf-DNA exhibited significantly higher copy numbers than mev-DNA across all FSMRs in Trisomy pregnancies, reinforcing its stronger methylation profile (Figures 4a–d).

### 3.6 Fold-change analysis of mcf-DNA and mev-DNA across trisomy and healthy pregnancies

Fold-change analysis comparing Trisomy pregnancies to healthy pregnancies revealed significant differences in mcf-DNA and mev-DNA for the *ERG* and *UMODL1 (U1* and *U2)* genes. For the *ERG* gene, mcf-DNA showed a substantially higher fold change (2.62-fold) than mev-DNA (1.53-fold; Supplementary Figure S1a). In the *UMODL1 (U1)* gene, both fractions showed similar alterations, with mcf-DNA and mev-DNA demonstrating 1.70-fold and 1.72-fold changes, respectively (Supplementary Figure S1b). In contrast, the *UMODL1 (U2)* gene showed a markedly greater change in mcf-DNA (3.31-fold) compared to mev-DNA (2.47-fold). These findings suggest that mcf-DNA exhibits a greater magnitude of change than mev-DNA for the *ERG* and *UMODL1 (U2)* genes (Supplementary Figure S1c).

## 4 Discussion

Early non-invasive detection of aneuploidy risk is crucial for prenatal care. In this study, we investigated the expression of FSMRs [*RASSF1A, ERG, UMODL1 (U1* and *U2)*] using qPCR on mcf-DNA and mev-DNA, which were isolated from blood plasma samples of non-pregnant women, healthy pregnant women, and women with Trisomy 21 pregnancies to assess trisomy risk. The results indicated that non-pregnant women have higher Ct values for both mcf-DNA and mev-DNA, indicating lower levels of methylated DNA due to the absence of fetal DNA. In contrast, healthy pregnant women have lower Ct values, signifying higher levels of methylated DNA compared to non-pregnant women. Women with Trisomy 21 pregnancies show even lower Ct values, indicating significantly higher methylated DNA levels compared to healthy pregnant women. The elevated methylated DNA levels in Trisomy 21 cases may be attributed to the presence of an extra chromosome, increased placental turnover, enhanced syncytiotrophoblast apoptosis, or altered extracellular vesicle secretion—all of which contribute to higher levels of free fetal DNA in maternal circulation (5, 13), which was subsequently detected through MeDIP-qPCR. This method refines and reproduces the differentiation between normal and Trisomy 21 pregnancies based on their methylation status.

Several studies have reported that cf-DNA levels increase during pregnancy due to the contribution of fetal DNA to the maternal circulation (19). Our results are consistent with these findings, showing higher levels of mcf-DNA in pregnant women compared to non-pregnant women. The association between sex chromosome aneuploidy and differential DNA methylation patterns has also been reported (20). Furthermore, Lim et al. (21) have found that tissue-specific DNA methylation signatures are associated with trisomy, suggesting that epigenetic markers could be used to distinguish between euploid and aneuploid pregnancies. The use of methylated DNA markers, particularly RASSF1A, has also been reported as a reliable indicator of fetal DNA in maternal plasma, as confirmed by methylation-sensitive restriction enzyme analysis (12).

However, a distinct aspect of our study is the comparative evaluation of methylation profiles between mcf-DNA and mev-DNA in maternal plasma. EVs, including exosomes and microvesicles, are known to carry genetic and epigenetic information that can be transferred between cells and may play a role in intercellular communication (22, 23). The analysis of ev-DNA has emerged as a promising area of research, as it may provide additional insights into pregnancy and aneuploidy. Furthermore, some studies have reported that abnormal changes in the concentration and composition of EVs and ev-DNA in maternal blood are associated with pregnancy-related complications, including pre-eclampsia (PE), gestational diabetes mellitus (GDM), preterm birth (PTB), and other adverse pregnancy outcomes (9, 24–26). Zang et al. (27) demonstrated that massively parallel sequencing of ev-DNA from maternal plasma can effectively distinguish between euploid and aneuploid pregnancies.

Our study utilized a methylation-specific approach focusing on FSMRs on chromosome 21 to enhance the detection of aneuploidies. This method significantly refines and reproduces our methodology for assessing the fetal genetic profile (11). We investigated methylation variations between normal and Trisomy 21 cases using MeDIP and qPCR to compare the methylation levels of mcf-DNA and mev-DNA derived from maternal plasma. Our results revealed that mcf-DNA exhibited higher methylation levels (reflected by lower Ct values) and increased copy numbers than mev-DNA, indicating a relatively higher abundance of fetal-specific methylated fragments. This observation reflects a quantitative difference between the DNA fractions. Zang et al. (27) reported no statistically significant difference in the coefficient of variation of relative read counts between ev-DNA and cf-DNA in maternal plasma samples. Furthermore, total ev-DNA exhibited higher GC content and mitochondrial DNA (mtDNA) percentages, while the fetal fraction of ev-DNA was significantly lower compared to cf-DNA. The underlying reasons for these discrepancies remain unclear. Pan et al. (28) further revealed that ev-cell-free DNA remains stable throughout gestation, while the fetal ev-DNA shows a slight decrease as gestational age increases. These findings are consistent with our study, suggesting that the reduced fetal fraction in EVs contributes to the lower sensitivity of ev-DNA for aneuploidy detection. This lower fetal fraction in ev-DNA may be directly linked to its reduced yield, which, in turn, might lead to lower methylation levels of mev-DNA compared to mcf-DNA in Trisomy 21 pregnancies. Moreover, variations in the size and concentration of EVs in maternal plasma may further influence the amount of ev-DNA available for analysis (24, 29).

However, Takur et al. (30) reported contrasting results, showing that the sensitivity of mutation detection in ev-DNA and cf-DNA was nearly equal across the four stages of colon carcinoma. Kahlert (31) reported that mutated cf-DNA, which is more tumor-specific in smaller fragments, predicts late-stage tumors, whereas ev-DNA serves as a better biomarker for early-stage detection. Furthermore, several studies support the effectiveness of ev-DNA in cancer research (32, 33). Next-generation sequencing of ev-DNA for common mutations, such as *BRAF*, *EGFR*, and *KRAS,* has been shown to be more sensitive than sequencing tumor DNA or cf-DNA from blood plasma (32). These findings suggest that mev-DNA could serve as an additional complementary marker. Even slight differences in the above findings may be attributed to the methodological differences in isolating and analyzing total EVs (29, 34). The combined analysis of mev-DNA and mcf-DNA may enhance the reliability and reproducibility of non-invasive prenatal testing, thereby facilitating earlier and more consistent detection of Trisomy 21. This may also reduce the need for invasive procedures such as amniocentesis and chorionic villus sampling (CVS), thereby lowering the associated risks and discomfort for pregnant women.

Employing advanced non-invasive epigenetic prenatal testing methods, as proposed in this study, could have significant economic and social implications. In India, where public non-invasive prenatal testing programs are lacking, introducing a sensitive and non-invasive method for detecting aneuploidies could help reduce the incidence of infants born with conditions such as Down syndrome (35). This, in turn, would lessen the long-term economic burden on the healthcare system and families. Furthermore, accurate and early detection of aneuploidies could minimize reliance on invasive procedures such as amniocentesis and CVS, which carry a risk of miscarriage. This non-invasive epigenetic approach would not only improve pregnancy outcomes but also enhance the overall quality of prenatal care (36, 37).

Despite some limitations, such as a small set of methylation markers (FSMRs), we obtained significant results. These findings compel us to further explore our findings in a larger cohort and in more diverse populations. Additionally, cf-DNA showed higher analytical performance than ev-DNA in Trisomy 21 detection, which may be due to the use of total EVs rather than placental alkaline phosphatase-positive exosomes (ExoPALP). Utilizing ExoPALP is expected to enhance the diagnostic potential of ev-DNA, while expanding the panel of methylation markers in future studies could further improve the robustness of the test.

## 5 Conclusion

Our study demonstrates that FSMRs, such as *RASSF1A, ERG, and UMODL1 (U1* and *U2),* can be detected in both cf-DNA and ev-DNA. They were isolated from maternal plasma using a MeDIP followed by qPCR approach in a North Indian cohort. Trisomy pregnancies exhibited higher expression of FSMRs in mcf-DNA and mev-DNA than healthy pregnancies. Notably, mcf-DNA showed greater feasibility and accuracy in detecting these epigenetic markers. These findings highlight the potential of both mcf-DNA and mev-DNA as reliable tools for non-invasive detection of aneuploidies in early pregnancies, especially in regions with limited access to advanced diagnostic technologies. Further validation in larger cohorts and more diverse populations is needed to enhance the sensitivity and specificity of this approach, making it more suitable for routine prenatal screening for chromosomal abnormalities. By advancing non-invasive prenatal testing, this research could improve clinical outcomes for feto-maternal health and offer significant economic and social benefits.

## Data Availability

The original contributions presented in the study are included in the article/Supplementary material, further inquiries can be directed to the corresponding author.
