# Supplementary material for: Early non-invasive epigenetic approach for assessing trisomy risk in maternal plasma
Source: Front Med (Lausanne). 2026 Mar 4;13:1704017. doi: 10.3389/fmed.2026.1704017 (PMC12997446; doi:10.3389/fmed.2026.1704017)
Supplement: Supplementary file 2 [file Image_1.pdf]

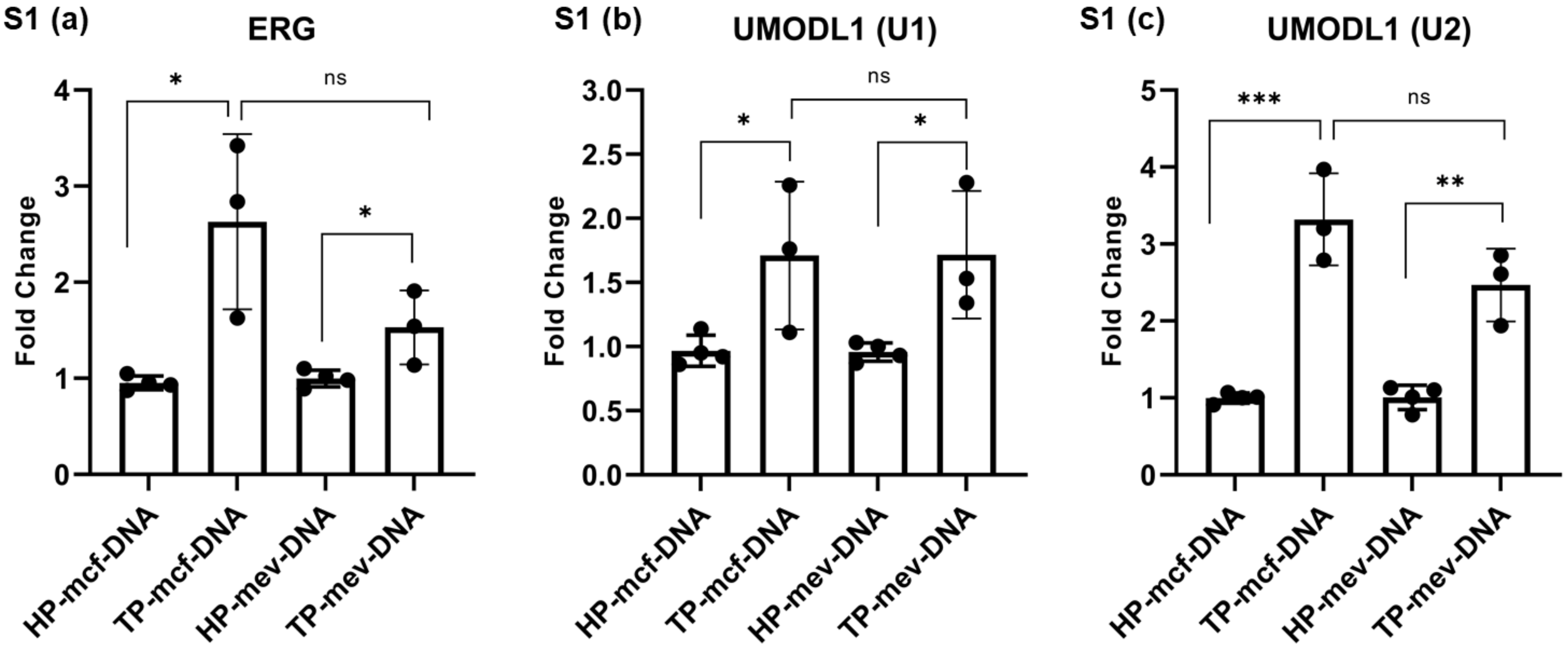

Figure S1: Fold-change analysis between healthy pregnancies (HP), women carrying trisomy pregnancies (TP), methylated cell-free DNA (mcf-DNA), and methylated extracellular vesicle-derived DNA (mev-DNA). **(a)** fold change of the ERG gene; **(b)** fold change of the UMODL1 (U1) gene; **(c)** fold change of the UMODL1 (U2) gene.
